# Evolution Analyses of CAMTA Transcription Factor in Plants and Its Enhancing Effect on Cold-tolerance

**DOI:** 10.3389/fpls.2021.758187

**Published:** 2021-11-01

**Authors:** Peixuan Xiao, Jia-Wu Feng, Xi-Tong Zhu, Junxiang Gao

**Affiliations:** Hubei Key Laboratory of Agricultural Bioinformatics, College of Informatics, Huazhong Agricultural University, Wuhan, China

**Keywords:** CAMTA transcription factors, phylogenetics analysis, gene expression, cold tolerance, gene regulatory network

## Abstract

The calmodulin binding transcription activator (CAMTA) is a transcription factor that is widely present in eukaryotes with conserved structure. It contributes to the response to biotic and abiotic stresses and promotes the growth and development of plants. Although previous studies have investigated the number and function of CAMTAs in some species, there is still a lack of comprehensive understanding of the evolutionary process, phylogenetic relationship, expression patterns, and functions of CAMTAs in plants. Here we identified 465 CMATA genes from 112 plants and systematically studied the origin of CAMTA family, gene expansion, functional differentiation, gene structure, and conservative motif distribution. Based on these analyses, we presented the evidence that CAMTA family was originated from chlorophyta, and we speculated that CAMTA might experience obvious structure variation during its early evolution, and that the number of CAMTA genes might gradually increase in higher plants. To reveal potential functions of CAMTA genes, we analyzed the expression patterns of 12 representative species and found significant species specificity, tissue specificity, and developmental stage specificity of CAMTAs. The results also indicated that the CAMTA genes might promote the maturation and senescence. The expression levels and regulatory networks of CAMTAs revealed that CAMTAs could enhance cold tolerance of rice by regulating carbohydrate metabolism-related genes to accumulate carbohydrates or by modulating target genes together with other transcription factors. Our study provides an insight into the molecular evolution of CAMTA family and lays a foundation for further study of related biological functions.

## Introduction

The divalent ion of calcium (Ca^2+^) is an ubiquitous second messenger in eukaryotes (Galon et al., 2010b), and it plays an important role in the growth and development of plants under biotic and abiotic stress (Kudla et al., 2010; Reddy et al., 2011). Calmodulins (CaM) is the most important type of Ca^2+^ receptor, and the Ca^2+^/CaM complex can bind to a large population of target proteins, including protein kinases, phosphatases, transcription factors, metabolic enzymes, ion channels, transporters, and molecular motors (Snedden and Fromm, 2001; Yang and Poovaiah, 2003; Bouché et al., 2005; Reddy et al., 2011; Poovaiah and Du, 2018). Currently, at least 90 transcription factors have been identified as CaM-binding proteins such as CAMTA, MYB, WRKY, NAC, bZIP, and MADS-box proteins (Reddy et al., 2002; Popescu et al., 2007; Kim et al., 2009; Galon et al., 2010b). Among these transcription factors, the calmodulin-binding transcription activator (CAMTA) is a conserved family and the most characteristic transcription factor related to calmodulin (Bouché et al., 2005; Finkler et al., 2007).

Tobacco ethylene response gene (*NtER1*) is the earliest CAMTA gene identified in plants. *NtER1* is highly expressed in senescent tobacco leaves and petals, suggesting it plays a regulatory role in tobacco development, and it is a trigger for senescence and death (Yang and Poovaiah, 2000). Six CAMTA genes (*AtCAMTA1-AtCAMTA6*) have been identified in *Arabidopsis*. They are highly responsive to environmental signals and stresses such as extreme temperature, ultraviolet radiation, salt, and injury, as well as hormones (Reddy et al., 2000; Galon et al., 2010a). For example, *AtCAMTA1*, *AtCAMTA2*, and *AtCAMTA3* work together to directly bind to the promoter of C-repeat binding factor (CBF2), thereby inducing CBF2 expression and improving the cold tolerance of plants (Doherty et al., 2009; Kim et al., 2013). *AtCAMTA1* also actively modulates drought response by regulating several stress response genes, including RD26, ERD7, RAB18, LTPs, COR78, CBF1, and HSPs (Pandey et al., 2013). As a plant immune negative regulator, AtCAMTA3 can regulate salicylic acid (SA)-mediated immune response by interacting with the *EDS1* gene promoter and inhibiting its expression (Du et al., 2009). Moreover, CAMTA homologues have been identified in some plants, such as the *LeER66* in tomato, the *BnCAMTA* in rape, and the *OsCBT* gene in rice (Zegzouti et al., 1999). A typical CAMTA protein has multiple characteristic domains, including a CG-1 domain, a TIG domain, an ankyrin (ANK) repeat domain, an IQ motif, and a CaM-binding (CaMB) domain. The CG-1 domain is a DNA-binding domain, while the TIG domain is involved in non-specific DNA binding. The ANK domain is involved in protein-protein interaction. The CaMB domain and a varying number of IQ motifs (IQXXXRGXXXR) bind to CaM in a calcium-dependent or calcium-independent manner, respectively (da Costa e Silva, 1994; Aravind and Koonin, 1999; Bork et al., 1999; Song et al., 2006).

In recent years, CAMTA genes have been idendified in various plants, such as *Z. mays* (Yue et al., 2015), *M. truncatula* (Yang et al., 2015), *V. vinifera* (Shangguan et al., 2014), *and G. max* (Wang G. et al., 2014). In addition, A previous study analyzed the evolution of CAMTA genes based on 35 plant genomes representing four major lineages and the 6 chlorophyta genomes available at that time (Rahman et al., 2016b). With more and more plant genomes deciphered, it is necessary to systematically analyze the CAMTA family from global perspectives. In this study, we analyzed the CAMTA genes from 112 plants, examined their gene structure and protein domain composition to reveal the origin and evolution of CAMTA, investigated the expression pattern in different tissues and different developmental stages, explored the role of CAMTAs in rice under low temperature stress. The study will provide a comprehensive insight into the evolution, expression, and function of CAMTA family, which might be conducive to further functional studies.

## Materials and Methods

### Data Collection

In this study, a total of 112 plant genomes were collected for subsequent analysis (Supplementary Table 1). Among them, a total of 64 plants were derived from the Phytozome database (Goodstein et al., 2012), 16 species from the Ensemble database (Hubbard et al., 2002), and 26 species from the NCBI database.^1^ Besides, *N. colorata* was collected from the National Genomics Data Center (Xue et al., 2021), *G. montanum* from the Dryad database,^2^ and *A. filiculoides* and *S. cucullata* from the Fernbase database.^3^

### Identification of Calmodulin Binding Transcription Activators From Plant Genomes

CAMTA protein sequences were integrated to establish a local protein database for identifying the CAMTA homologous sequences. The sequences of six *Arabidopsis* CAMTA protein were downloaded from the *Arabidopsis* Information Resource database^4^ (Lamesch et al., 2012), including AT1G67310.1, AT2G22300.1, AT3G16940.1, AT4G16150.1, AT5G09410.2, and AT5G64220.1. BLASTP (v2.10.0) was used to perform sequence alignment, *e*-value was set as 1e-10. The *Arabidopsis* CAMTA protein sequences were used as queries to align with the constructed protein database. Only the longest transcript from genes containing multiple transcripts was retained, and the shorter transcripts were removed. Then, four conserved domains or motifs of proteins including CG-1 domain, TIG domain, ANK repeats, and IQ motifs were identified by using three databases, namely, CDD, SMART, and PFAM, and the CaMB domain was identified using the Calmodulin target database. Only the sequences with these identified domains were defined as CAMTA protein.

### Subcellular Localization and Gene Ontology Annotation

The subcellular localization of all CAMTA protein sequences was performed using WoLF PSORT^5^ (Horton et al., 2007). Omics Box (v1.4.12)^6^ was employed for gene ontology annotation of the CAMTA family. First, protein sequences of the identified CAMTA genes were imported into Omics Box. Then, the gene functional annotation was conducted with default parameters. Finally, the results were visualized by using matplotlib (v3.3.3) (Hunter, 2007).

### Phylogenetic Analysis of Calmodulin Binding Transcription Activator Proteins

CAMTA protein sequences were aligned using MAFFT (v7.453) (Katoh and Standley, 2013), which is an efficient multi-sequence alignment software. ProtTest (v3.4.2) (Darriba et al., 2011) was used to select the best fitting model of protein evolution according to the AIC and BIC indicators, and the optimal amino acid substitution model was JTT+I+G. The phylogenetic tree based on the maximum likelihood method (bootstrap value = 1,000) was constructed by IQtree2 (v2.0.6) (Minh et al., 2020) and RaxML-HPC BlackBox of CIPRES (v3.3) (Miller and Schwartz, 2011), respectively. Finally, the tree was trimmed by using Interactive Tree Of Life (iTOL v5)^7^ (Letunic and Bork, 2019).

### Structure Analysis of Calmodulin Binding Transcription Activator Genes

The information of untranslated region (UTR), coding sequence (CDS), and intron phase was extracted and transformed into bed format with the GFF3 files of 112 plants as the input. The structure of CAMTA genes was visualized using Gene Structure Display Server (GSDS v2.0)^8^ (Hu et al., 2015). The phylogenetic tree of the CAMTA protein and the structure of CAMTA genes were displayed in the same figure. Since the lengths of the introns of some genes were too long to be displayed, the lengths of the introns were set as the same size. The phase and the number of the introns were also analyzed and displayed in the gene structure.

### Analysis of Calmodulin Binding Transcription Activator Genes Expression

The transcription data were from the NCBI SRA database^9^ and TraVA database^10^ (Supplementary Table 2). To compare the expression of the CAMTAs in multiple plants, we collected RNA-Seq data in *B. vulgaris*, *B. distachyon*, *C. quinoa*, *E. grandis*, *G. raimondii*, *H. annuus*, *M. domestica*, *O. sativa*, *P. trichocarpa*, *S. lycopersicum*, *V. vinifera*, and *Z. mays*, in which most samples derived from the leaves. In order to compare the expression of the CAMTAs in different tissues of the same plant, RNA-Seq data were sampled from different tissues in *O. sativa*, *Z. Mays*, and *A. thaliana*. The RNA-Seq data under low temperature stress were collected to investigate the expression of the CAMTA genes. The RNA-Seq data were converted into fastq format using SRA Toolkit (v2.10.4) (Leinonen et al., 2011) and filtered using Trimmomatic (v0.38) (Bolger et al., 2014), respectively. Then Hisat2 (v2.1.0) (Kim et al., 2019) was used to construct the genome index and perform read mapping, and RSEM (v1.3.3) (Li and Dewey, 2011) was used to quantify the CAMTAs. The expression heatmap was drawn using pheatmap in R package (v1.0.12) (Kolde, 2012).

### Genome-Wide Identification of Calmodulin Binding Transcription Activator Target Genes and Differentially Expressed Genes

The frequency matrix of *Arabidopsis* CAMTA motifs was downloaded from JASPAR database^11^ (Fornes et al., 2019), and MEME FIMO (v5.1.1) (Grant et al., 2011) was used to predict CAMTA target genes in the whole genome. Under cold stress, differentially expressed genes (DEGs) were identified by the tool DESeq2 (v1.26.0) (Love et al., 2014) and edgeR (v3.32.1) (Robinson et al., 2009) with fold change set as 2, and false discovery rate (FDR) set as 0.01. The intersection between DEGs and potential target genes detected by MEME were designated as target genes regulated by CAMTA. GENIE3 (v1.8.0) (Huynh-Thu et al., 2010) was used to analyze the regulatory effect of CAMTA genes on target genes, the weight threshold was set as 0.25. Gephi (v0.9.2) (Bastian et al., 2009) was used to visualize the regulatory network.

## Results

### Identification of Calmodulin Binding Transcription Activator Genes

**FIGURE 1:**
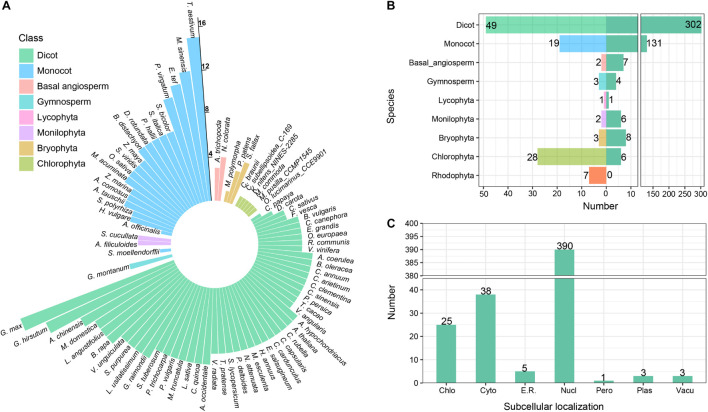
Identification and annotation of CAMTA genes. **(A)** Number of CAMTA genes in each species. The length of the bar represents the number of CAMTA genes in plant. **(B)** Number of representative species and identified genes. The left panel represents the number of species, and the right panel represents the number of identified genes. **(C)** Subcellular localization of identified CAMTA proteins. Chlo, chloroplast; Cyto, cytosol; E.R., Endoplasmic reticulum; Nucl, nucleus; Pero, peroxisome; Plas, plasma membrane; Vacu, vacuolar membrane.

The homologous sequences of CAMTA genes were retrieved from the constructed local database using BLASTP with protein sequences of six *Arabidopsis* CAMTA genes as queries. The longest transcript from the genes containing multiple transcripts was retained, and the redundant sequences were removed. Then, the conserved domains were manually validated against CDD, SMART, PFAM, and Calmodulin Target Database. After the strict screening, a total of 465 CAMTA protein sequences were obtained from 112 plants (Figures 1A,B and Supplementary Table 3). These representative plants included rhodophyta, chlorophyta, bryophyta, monilophyta, lycophyta, gymnosperms, and angiosperms, among which dicots exhibited the most abundant, followed by chlorophyta. None of the 7 rhodophyta species was found to contain CAMTA protein sequence. CAMTA protein sequences were identified from only 6 out of 28 chlorophyta plants, and they were single copies, implying that CAMTA might be originated from chlorophyta.

A total of 131 CAMTA genes were identified from 19 monocots with 4 or more CAMTAs from each monocot except *A. officinalis*. Among them, the number of CAMTA genes from *T. aestivum*, *M. sinensis*, *E. tef*, and *P. virgatum* was 15, 12, 11, and 10, respectively. In total, 302 CAMTAs were identified from 49 dicots with 4 or more from each dicot except 1 from *C. papaya*, 2 from *D. carota*, 3 from *C. sativus*, and *F. vesca.* In addition, 15, 13, and 10 CAMTAs were obtained, respectively, from *G. max*, *G. hirsutum*, and *A. chinensis*. For basal angiosperm, 4 CAMTAs were identified from *N. colorata* and 3 from *A. trichopoda*. For gymnosperm, 4 CAMTAs were obtained with all of them from *G. montanum*. However, no complete CAMTA genes were identified from *P. taeda* and *P. abies*. For Lycophyta, only 1 CAMTA gene was identified from *S. moellendorffii*. For Monilophyta, 3 CAMTA genes obtained from *A. filiculoides* and *S. cucullata*, respectively. For Bryophyta, 1 CAMTA gene was identified from *M. polymorpha*, 3 from *P. patens*, and 4 from *S. fallax*.

Based on the above results, we found that the CAMTA genes did not exist in rhodophyta, but they appeared in some chlorophyta plants and evolutionarily late land plants. Considering that most chlorophyta plants live in fresh water, and their living environments are greatly different from those of rhodophyta in seawater, the appearance of new genes might contribute to chlorophyta’s adaptation to the new environment. Therefore, we speculated that the CAMTA genes might be originated from some chlorophyta plants, which is consistent with the previous research results (Zhang et al., 2020). In our study, the number of CAMTA genes varied greatly in different phyla and exhibited a trend of gradual increase during evolution process, indicating many gene duplication events in the long-term evolution of CAMTA genes. In higher angiosperm, the copy number of CAMTAs varied greatly across different species. *G. max* had the maximum copies (up to 15), followed by *G. hirsutum* with 13 copies, while *A. officinalis*, *C. papaya*, and *C. sativus* had only 1∼3 copies. Moreover, no obvious pattern of CAMTA number distribution was found in the species under the same class. This showed that the evolution of CATMAs was a very complex process under the influence of multiple factors, thus the distribution of CAMTA number cannot be explained merely from the perspective of a single factor. For example, plants suffered various biotic and abiotic stresses from aquatic to terrestrial environment. As a transcription factor playing important roles in stress response, CAMTA was bound to be affected. In addition, genome-wide duplication events and chromosome deletion might also lead to the change in CAMTA gene number.

Subcellular localization information provides an important clue for exploring protein function. WoLF PSORT was used to analyze the subcellular localization of the identified CAMTA proteins (Figure 1C). The results showed that 390 CAMTA proteins were located in the nucleus, accounting for 83.87% of all the sequences; 38 CAMTAs were located in the cytosol, accounting for 8.17%; 25 CAMTAs were located in chloroplast, accounting for 5.37%. The remaining CAMTAs were located in the endoplasmic reticulum, peroxisome, plasma membrane, and vacuolar membrane, accounting for 2.59% in total. CAMTA proteins were mostly located in the nucleus, which was in accordance with the transcriptional activation function of CAMTA as a transcription factor. Some CAMTAs contain nuclear localization signal (NLS) sequence, thus they can enter the nucleus to perform their function. For example, in *M. truncatula*, all of the MtCAMTA proteins were predicted to contain an NLS at the N-terminus of the protein, which was consistent with their function in the nucleus as transcription factor (Yang et al., 2015). In *B. napus L.*, all the BnCAMTA proteins were also predicted to contain an NLS at the N-terminus of the protein (Rahman et al., 2016a).

### Phylogenetic Analysis of Calmodulin Binding Transcription Activator Protein Sequences

**FIGURE 2:**
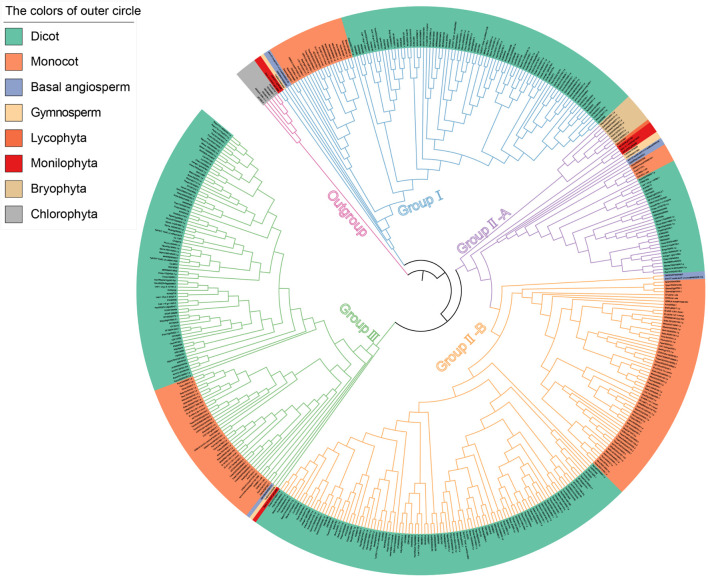
Phylogenetic tree based on the identified CAMTAs. The different colors of the clades represent different groups. The background colors of the outer circle represent different species.

In order to explore the phylogenetic relationships of CAMTA families, a phylogenetic tree of CAMTA proteins was constructed based on the maximum likelihood (Figure 2). The six CAMTA proteins of chlorophyta were used as the outgroup and root node. The 459 protein sequences can be grouped into three clades, namely, Group I, Group II, and Group III. The base nodes of each group represented ancient plants such as bryophyta or monilophyta, followed by basal angiosperm, monocot, and dicot, which was in line with the evolutionary process of plants. In addition, the plants were regularly clustered together by class in each group. Therefore, from the perspective of evolution and clustering, our phylogenetic tree was reasonable. Each group was originated from lower plants, and it contained bryophyta or monilophyta, gymnosperm, and angiosperm, indicating the differentiation of CAMTA protein in early evolution stage of land plants. Considering the great differences among sea water, fresh water, and land environment, we speculated that the adaptation to the new living environment might contribute to this differentiation. These differentiations were maintained during the long evolutionary process, thus a large number of variations were accumulated in the sequence. The largest clade Group II consisted of two subgroups, which were termed as II-A and II-B. As shown in Figure 2, both II-A and II-B contained basal angiosperm, monocot, and dicot, while there were more ancient species in II-A, including bryophyta, monilophyta, lycophyta, and gymnosperm. These results suggested that subgroup II-B might diverge from subgroup II-A in basal angiosperm. Although II-B was originated later than II-A, it contained the largest number of species, which exceeded Group I and Group III, indicating that the subgroup experienced rapid gene expansion. Although there were no obvious subgroups in Group I and Group III, these two groups had large number of monocot and dicot species, indicating that their protein sequences constantly evolved and their number expanded overall. The variation of sequence composition in each group also promoted the change of protein domain. Thus we further analyzed protein domain in the following section.

To sum up, we inferred that CAMTA was originated from chlorophyta, and then differentiated into three groups, and CAMTAs expanded rapidly to be adapted to the great changes from aquatic to land environment. In particular, the largest clade Group II underwent great variation and differentiated into two subgroups. The available data support that differentiation events occurred in basal angiosperm.

### Structure Analysis of Calmodulin Binding Transcription Activator Genes

**FIGURE 3:**
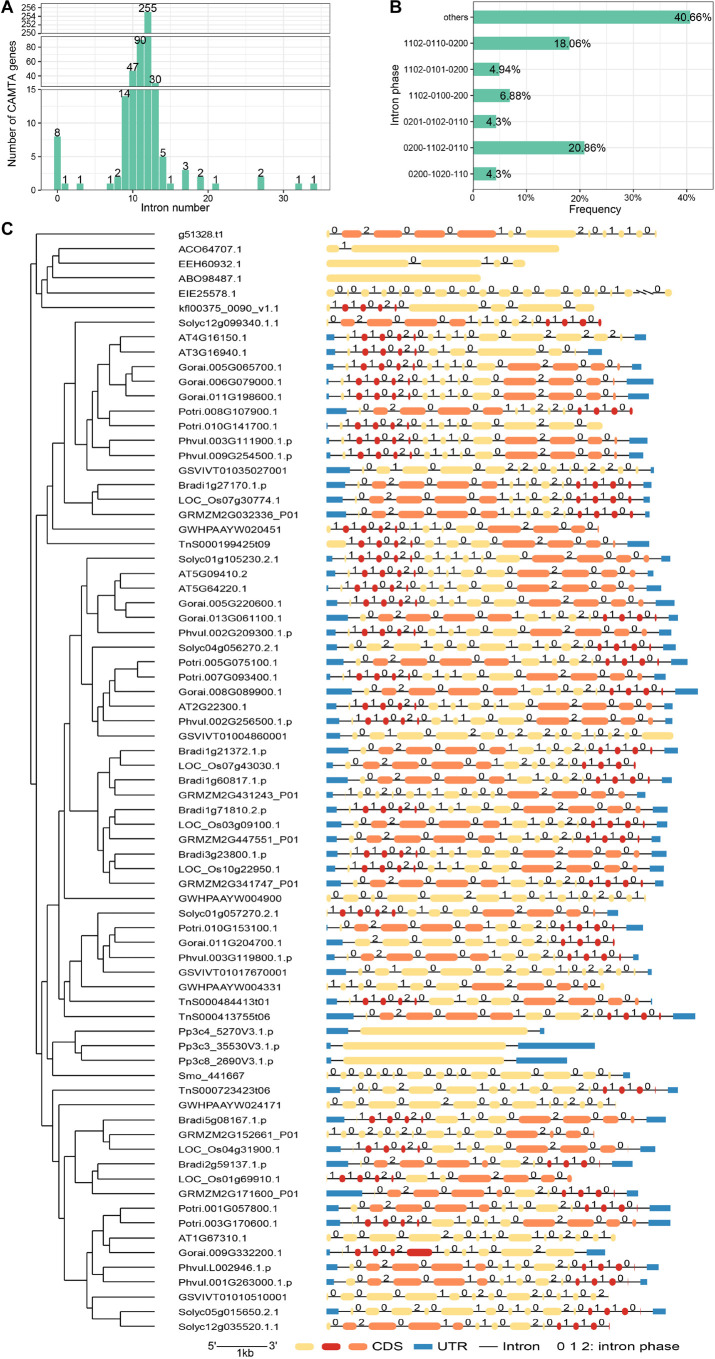
Gene structure analysis of CAMTAs. **(A)** Distribution of intron number. **(B)** Percentage of intron phase. **(C)** CAMTA structures of some representative species. Pink indicates the exons corresponding to intron phase pattern 0200 and 0201, and dark red denotes those corresponding to intron phase pattern 1102 or 0110.

Knowledge of the intron phases helps to understand the evolution of the gene structure and alternative splicing of CAMTA genes. Introns can be divided into three types based on phases: phase 0, phase 1, and phase 2. Phase 0 intron does not disrupt a codon, and phase 1 intron disrupts a codon between the first and second bases, whereas phase 2 intron disrupts a codon between the second and third bases (Choudhuri, 2014). The intron phase can affect exon translation. In this study, we counted the number of introns of the CAMTA genes and found that the number of introns in CAMTA genes varied greatly across species, ranging from 0 to 34 (Figure 3A and Supplementary Table 4). Among our identified CAMTA genes, *O. lucimarinus CCE9901*, *P. patens*, and *S. fallax* contained no introns, which was consistent with a previous report (Rahman et al., 2016b). This indicated that CAMTA experienced significant structure variation during its early evolution. The number of introns in other plants was mostly between 10 and 13. In 255 genes, each gene contained 12 introns, accounting for 54.83%, and in other 90 genes, each gene contained 11 introns, accounting for 19.35%. In most cases, the same species and clade had similar exon-intron structure and intron number. Only a small number of CAMTA genes showed various exon-intron structure and intron number with more than 20 introns contained in each gene, which was much higher than the average of 10∼13. For example, Dr04389.1.cds had 34 introns, and MDP0000265800 had 21 introns. Overall, we discovered that the number of introns in most of the same species varied slightly, indicating that the gene structure was relatively conserved in the same species. The conservation of CAMTA structure implied that CAMTA was related to important functions of life activities.

The analysis of the intron phases indicated that intron phases exhibited certain distribution patterns (Figure 3B). Pattern 0200-1102-0110 and pattern 1102-0110-0200 accounted for 20.86 and 18.06%, respectively, far more than other patterns. Pattern 1102-0101-0200, 1102-0101-0200, 0201-0102-0110, and 0200-1020-110 accounted for 6.88, 4.94, 4.30, and 4.30%, respectively. The exon-intron structure of CAMTA was visualized by GSDS (Figure 3C and Supplementary Figure 1). The representative species were selected to basically cover the evolution process of plants, including chlorophyta, bryophyta, monilophyta, gymnosperm, basal angiosperm, monocot, and dicot plants (Supplementary Table 5). Interestingly, we noticed that the length of exons and the phase of introns showed a correlation to a certain extent. As shown in Figure 3C, the intron phase pattern 0200, 0201, and 1102 were abundant in CAMTA genes. The lengths of the exons corresponding to intron phase pattern 0200 or 0201 (pink) were significantly longer than those corresponding to 1102 or 0110 (dark red).

### Domain Analysis of Calmodulin Binding Transcription Activator Proteins

We identified a total of 465 CAMTA protein sequences and calculated their length, molecular weight, and isoelectric point (Supplementary Table 6). The average length of CAMTA proteins was 1004.5 aa. Protein PHT66961 (from *C. annuum*) was the shortest with a length of 745 aa and a molecular weight of 84.2 kDa. Protein Dr04389.1.cds (from *D. rotundata*) was the longest with a length of 2309 aa and a molecular weight of 254.4 kDa. The maximum, minimum, and average of isoelectric points were 9.34, 5.02, and 6.55, respectively. Using CDD, SMART, PFAM, and Calmodulin Target Database, we identified CG-1 domain, TIG domain, CaMB domain, ANK repeats, and IQ motifs.

**FIGURE 4:**
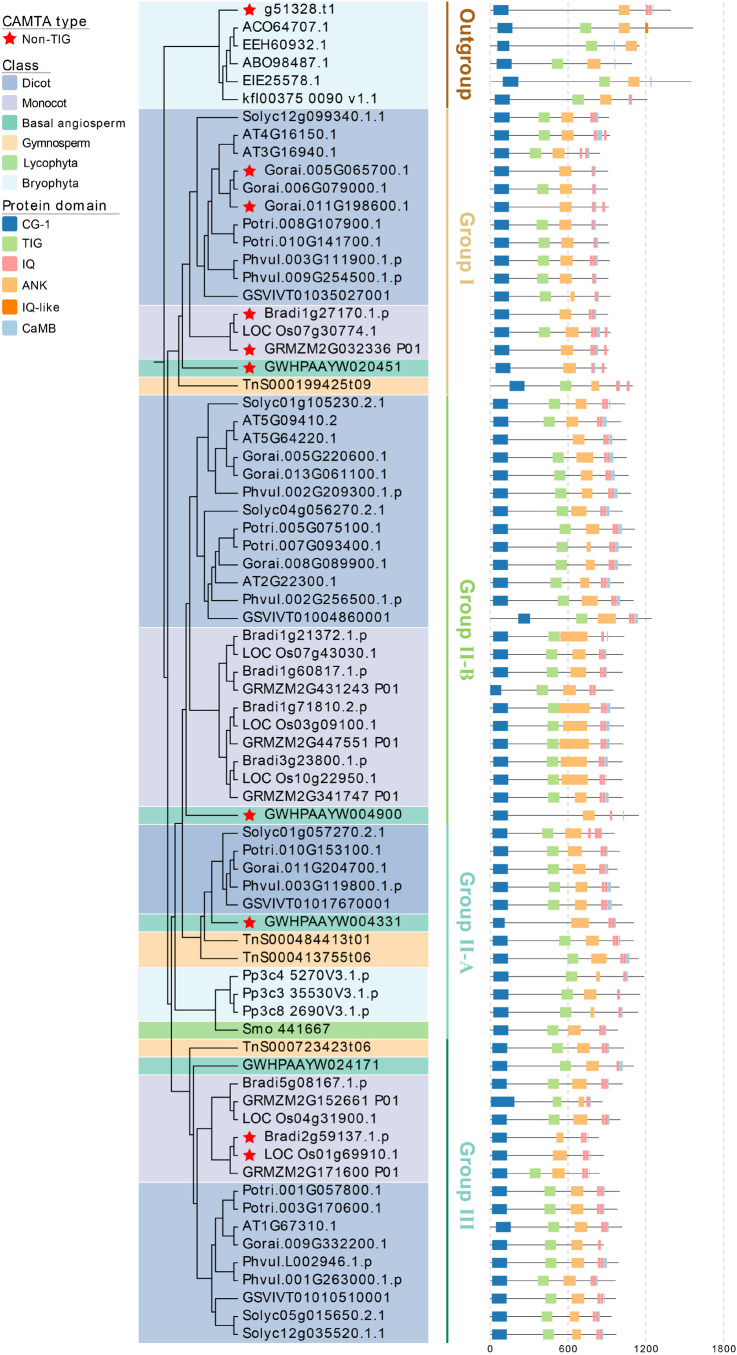
Phylogenetic tree and functional domains of CAMTAs in representative species. CG-1, CG-1 DNA-binding domain; TIG, transcription-associated immune globulin-like domain; ANK, ankyrin repeat domain; IQ, Ca^2+^-independent CaM-binding IQ motif; CaMB, Ca^2+^-dependent CaM binding domain.

Although the CAMTA proteins contained very conserved domains, the distribution patterns of these domains varied among the species and the groups (Figure 4 and Supplementary Figure 2). As described above, the selection criterion of the representative proteins was similar to those of the representative species (Figure 4 and Supplementary Table 5). Generally, CAMTA proteins included CG-1 DNA-binding domain, TIG domain involved in non-specific DNA binding, ankyrin repeats, IQ motifs of Ca^2+^-independent calmodulin-binding domain, and Ca^2+^ dependent calmodulin-binding domain. All the CAMTA proteins contained ANK repeats except MDP0000255517. The number of ANK repeats was quite different, leading to their significantly different length. The number of IQ motifs contained in CAMTA proteins varied from 0 to 3, and this variation was slightly weaker than that of ANK repeats. Three IQ motifs were contained in each of 15 CAMTA proteins, two in each of 356 CAMTA proteins, and one in each of 89 CAMTA proteins, and no IQ motif in 3 CAMTA proteins. In addition, two CAMTA proteins contained IQ-like motif.

Some CAMTA proteins contained TIG domain, while others did not. According to the presence and absence of TIG domain, the CAMTA protein was divided into TIG-CAMTA and non-TIG CAMTA (Rahman et al., 2016b), and their distribution patterns were different across phylogenetic clusters and species. Considering this, we analyzed the TIG domain of each species, and found a total of 401 TIG CAMTAs and 64 non-TIG CAMTAs. In all the chlorophyta species except *C. braunii*, the CAMTAs fell into TIG ones. In bryophyta except for two CAMTA proteins from *S. fallax*, all the other CAMTAs were TIG CAMTAs. In monilophyta, lycophyta, and gymnosperm, all the CAMTA proteins were TIG CAMTAs. These results indicated that non-TIG CAMTAs might originate from the late-stage chlorophyta, and they persisted in early land plants from bryophytes to angiosperms. Moreover, the distribution of TIG CAMTAs and non-TIG CAMTAs between monocots and dicots, as well as among group I, II, and III were uneven. As shown in Supplementary Figure 2, among the 302 CAMTAs of the monocots, the proportions of TIG, and non-TIG CAMTAs were 87.09 and 12.91%, respectively; among the 131 CAMTAs of dicots, the proportion of TIG and non-TIG were 77.86 and 22.14%, respectively. Group III had only 3 non-TIG CAMTAs, which were far less than those from group I and II. In group I, there were 21 CAMTAs from monocots, of which 17 were non-TIG, and 84 CAMTAs from dicots, of which 21 were non-TIG. Only a few non-TIG CAMTAs were identified from the two subgroups of group II. Most non-TIG CAMTAs in group II-B were from dicots, whereas non-TIG CAMTA in group II-A were not found in the dicots. The difference in protein domain distribution of CAMTAs suggested the differentiation of their function.

### Expression Analysis of Calmodulin Binding Transcription Activator Genes

**FIGURE 5:**
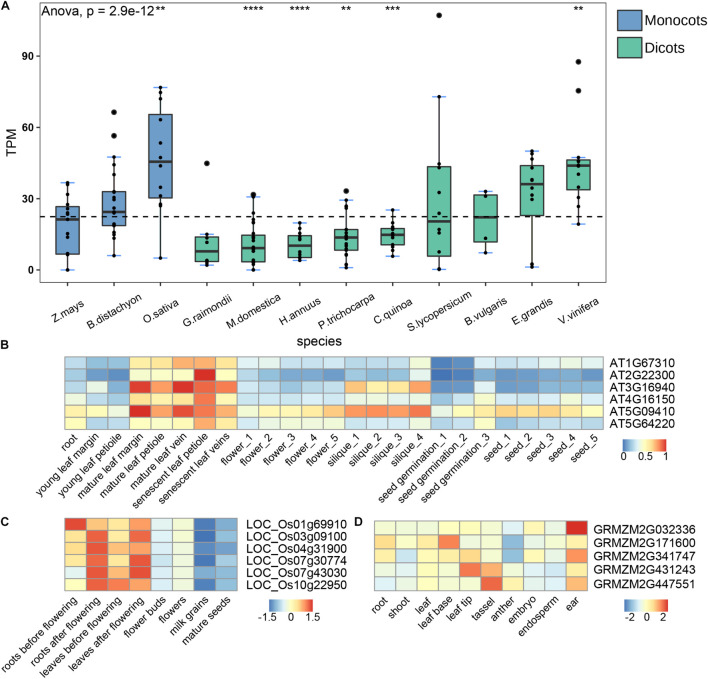
Expression analysis of the CAMTA genes. **(A)** RNA-seq analysis of different species. The dotted line represents the average gene expression. Box plots indicate the first quartile, the median, and the third quartile. TPM indicates Transcripts Per Kilobase of exon model per Million mapped reads. **(B–D)** CAMTA expression in different tissues of *A. thaliana*, *O. sativa*, and *Z. mays*. The numbers behind the tissues represent different developmental stages. The symbols of “*”, “**”, “***”, and “****” represent the *p*-values are 0.05, 0.01, 0.001, and 0.0001, respectively.

Gene expression profiles were closely related to their functions. To better understand the functions of CAMTA genes, we analyzed the transcription patterns of multiple representative species including *B. vulgaris*, *B. distachyon*, *C. quinoa*, *E. grandis*, *G. raimondii*, *H. annuus*, *M. domestica*, *O. sativa*, *P. trichocarpa*, *S. lycopersicum*, *V. vinifera*, and *Z. mays* (Supplementary Table 2). In order to compare gene expression between different species, we collected RNA-seq data from the same tissue. Most data were from leaves except that *C. quinoa* data derived from whole seeds and *P. trichocarpa* data from stems. We calculated the transcripts per million (TPM) of each CAMTA gene for every species, and then computed the average of TPM to represent the expression level according to the number of CAMTA genes in the species. The results showed that the expression level had significant species specificity (Figure 5A). The expression levels of the CAMTA genes in *O. sativa* and *V. vinifera* were significantly higher than the average, while those in *P. trichocarpa*, *H. annuus*, and *M. domestica* were significantly lower than the average. No obvious expression pattern was observed in dicot and monocot. In addition, Group II-B CAMTAs were identified from all the 12 plants, and their expression levels were generally high. However, group II-A genes were only identified from 5 plants, and their expression levels were generally low except in *V*. *vinifera* (Supplementary Figure 3).

In order to explore the expression differences of CAMTA genes in different tissues, we collected transcription data from multiple tissues for further analysis, including 25 tissues of *A. thaliana*, 8 tissues of *O. sativa*, and 10 tissues of *Z. mays* (Supplementary Table 2).

The expression levels of CAMTA family showed significant tissue specificity, that is, the same tissue generally clustered together, but different tissues had significant differences (Figure 5B). For example, the expression level of AT3G16940 in mature leaves and siliques was much higher than that in other tissues, and that of AT5G64220 was significantly lower in seeds and young leaves. The expression level of CAMTAs also showed the specificity of developmental stage. They were significantly higher in mature and senescent leaves than in young leaves. Previous studies have shown that PtCAMTA1 is highly expressed in mature leaves of *P. tomentose*, and that some CAMTA genes in tomato show high expression in fruits, indicating that CAMTA genes are closely related to fruit development and maturity (Yang T. et al., 2012; Wei et al., 2017), which are basically consistent with ours. Therefore, we speculated that the CAMTA genes might promote the maturation and senescence of *A. thaliana*.

To examine whether the results of CAMTA genes from *A. thaliana* are applicable to other model plants, we compared the expressions of CAMTAs in roots, leaves, flowers, and seeds of *O. sativa* at different developmental stages and found significant specificity. As shown in Figure 5C, the expression levels of almost all CAMTA genes in leaves after flowering were substantially higher than those before flowering. Similar results were observed between roots after flowering and before flowering, between flowers and flower buds, between mature seeds and milk grains. Overall, the expression levels of CATMA genes were higher in late developmental stage than in early developmental stage. These results further support the role of CAMTA in promoting maturation. We also analyzed RNA-seq data collected from the root, shoot, leaf, leaf base, leaf tip, tassel, anther, embryo, endosperm, ear of *Z. mays*, and obtained the results of tissue specificity, which were similar with our findings in *A. thaliana* (Figure 5D). For example, the expression level of GRMZM2G171600 was the highest in leaf base, but the lowest in anther. In brief, the expression level of CAMTA genes exhibited significant species specificity, tissue specificity, and developmental stage specificity. The CAMTA genes might promote the maturation and the senescence of plants.

### Function Analysis of Calmodulin Binding Transcription Activator Genes in Rice Under Cold Stress

**FIGURE 6:**
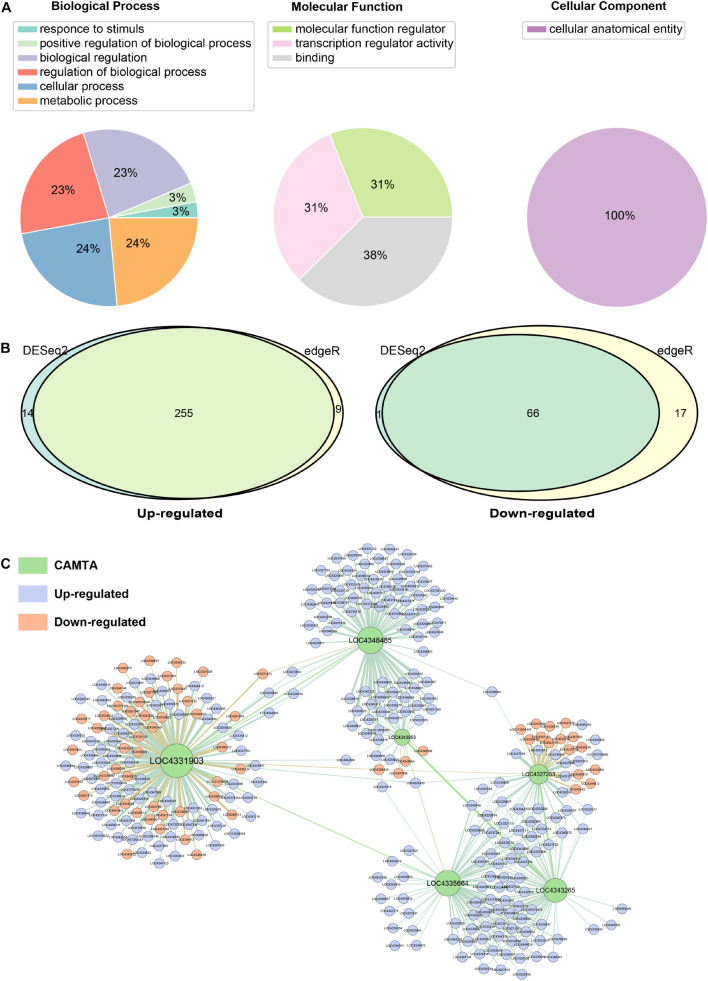
Function analysis of the CAMTA genes. **(A)** Gene Ontology (GO) analysis of identified CAMTA genes. GO analysis of CAMTA protein sequences mainly in terms of biological process, molecular function, and cellular component. **(B)** Analysis of differential expression in rice under cold stress by using DESeq2 and edgeR. **(C)** Regulatory network of CMATA genes in rice under cold stress.

The Gene Ontology (GO) database was used to analyze molecular function (MF), cellular component (CC), and biological process (BP) of identified CAMTA genes (Figure 6A). The annotation assigned these CAMTA genes into six biological process categories, and most of these genes were involved in regulation of biological process, cellular process, and metabolic process. In the molecular function, CAMTA genes were enriched in molecular function regulator (31%), transcription regulator activity (31%), and binding (38%). In the cellular component, we found that all the CAMTA genes were enriched in cellular anatomical entity (Figure 6A).

Low temperature is one of the main environmental stresses that plants frequently experience under natural conditions. As a transcription factor, CAMTA has a variety of functions, and its role in promoting maturation was described in the previous sub-section. We further investigated its role in resisting low temperature stress. We used two sets of RNA-Seq data including PRJNA430015 and PRJNA607661 (Supplementary Table 2), with or without cold stress to explore the function of rice CAMTAs and their target genes. DESeq2 and edgeR were separately used for differential expression analysis. By integrating two results, a total of 255 CAMTA target genes were found to be up-regulated, and 66 down-regulated (Figure 6B).

DAVID was used to annotate the differentially expressed target genes (Supplementary Table 7). The results showed that some down-regulated genes were involved in carbohydrate metabolic process, such as beta-glucosidase 20/26 (LOC9270758/LOC4344146) and glucan endo-1, 3-beta-glucosidase GII (LOC4326519). Some other down-regulated genes were involved in ATP binding, such as serine/threonine-protein kinase Nek3/SAPK3 (LOC4349411/LOC4342542) and UMP-CMP kinase 1 (LOC4339882). Some up-regulated genes were involved in transcriptional regulation, protein catabolism, and defense response regulation. In addition, we found that the AP2/ERF transcription factor gene family was up-regulated, including dehydration responsive element-binding protein 1C (LOC4339974) and ethylene-responsive transcription factor 5/7/11 (LOC4336947, LOC4349395, LOC4337341). Among them, dehydration responsive element-binding protein 1C is also called transcription factor CBF1, which can bind to GCC-box elements to induce the expression of cold stress-related genes. In addition, there were some other cold resistance-related transcription factors, such as NAC, MYB, bZIP, and WRKY (Liu et al., 2012; Yang A. et al., 2012; Wang L. et al., 2014; Diao et al., 2020). We also constructed regulatory network of six CAMTA genes in rice using GENIE3 and found various regulatory patterns (Figure 6C). First, LOC4331903 had the most targets (123 genes), while LOC43953 had the least targets (only 33 genes). Second, LOC4331903, LOC4327253, and LOC4343953 had both up-regulated and down-regulated target genes, but the target genes of LOC4335664 and LOC4343265 were all up-regulated. These results suggested that the regulation roles and patterns of the CAMTAs under cold stress have differentiated, and that LOC4331903 might have more important regulatory functions and more complex regulatory patterns than other CAMTAs. In addition, we also found that some target genes were regulated by both LOC4343953 and LOC4348485, and some target genes by three CAMTAs, namely, LOC4327253, LOC4335664, and LOC4343265. However, LOC4331903 shared very few target genes with other CAMTAs, namely, this CAMTA independently regulated target genes. This implied that LOC4327253, LOC4335664, and LOC4343265 might jointly regulate their target genes, LOC4343953 and LOC4348485 tended to cooperate, but LOC4331903 was more likely to function independently.

On the one hand, CAMTA may increase the cold resistance of plants by regulating the expression of carbohydrate-related genes to accumulate more carbohydrate. On the other hand, under cold stress, CAMTA transcription factors can regulate the transcription of target genes or work together with other transcription factors to increase rice resistance to low temperature stress.

## Discussion

### Origin of Calmodulin Binding Transcription Activator Genes

Although genome-wide identification of CAMTA family has been completed in *A. thaliana*, *O. sativa*, *Z. mays*, *G. max*, *M. truncatula*, and other plants, the origin of CAMTA is still a controversial issue. A previous study identified the CAMTA genes from 6 chlorophyta genomes available at that time and 35 plant genomes, and found that no gene containing both a CG-1 domain and an IQ domain or a CaMB domain in any chlorophyta genome (Rahman et al., 2016b). Based on these findings, CAMTA genes were assumed to emerge from the embryophyta lineage ancestor. As mentioned in the Introduction section, the CAMTA proteins consist of multiple functional domains or motifs, including CG-1, TIG, ANK, IQ, and CaMB. Among them, the CG-1 is a unique DNA-binding domain, which is necessary for CAMTA to bind to DNA and perform its function. The IQ motifs can bind to CaM and CaM-like proteins in a Ca^2+^-independent manner. Similar to the IQ motifs, CaMB domain is also a calmodulin-binding domain, but it binds to CaM and CaM-like proteins in a Ca^2+^-dependent manner. In the previous study, only six chlorophyta genomes were used, in which no gene contained both CG-1 and IQ motif or CaMB domain simultaneously. In our study, CAMTA genes were undetectable in rhodophyta, but they were identified from 6 out of 28 chlorophyta plants. All these identified CAMTA genes harbored the CG-1 domain, the ANK domain, and at least one of CaMB domain, IQ, or IQ-like motif (Figure 4). The results showed that except that CaMB domains of our identified chlorophyta CAMTAs were slightly smaller than those of land plant CAMTAs, there was no significant difference in CAMTA genes between chlorophyta and land plants. However, the size of CaMB domains identified from chlorophyta were similar to that in some CAMTAs from basal angiosperms and lycopodiopsida species, such as GWHPAAYW020451, GWHPAAYW004900, and Smo 441667. The CaMB domains with similar size could also be found in the some CAMTAs of even a few monocots and dicots, such as Gorai.006G079000.1, Solyc01g105230.2.1, LOC Os10g22950.1, and LOC Os01g69910.1. These genes have the functional domains of CAMTA, especially characteristic CG-1, CaMB, or IQ, thus we speculated that CAMTAs might be originated from chlorophyta. CAMTAs were detected from a small number of chlorophyta and all the evolutionarily young land species, but not from the evolutionarily old rhodophyta, which confirmed our speculation. More importantly, a recent study has reported the evolution and diversity of transcription factors in *A. angustus* and 18 green plants, and has identified CAMTAs from *K. nitens* and *C. braunii* (Zhang et al., 2020). *K. nitens* belongs to the nematophyta order of chlorophyta, and usually grows in filamentous form in temperate streams (John, 2003). *C. braunii* is an evolutionarily advanced chlorophyta species, and it resembles a land plant. These evidences also support our speculation that CAMTA is originated from chlorophyta. In addition, some studies have indicated that many gene families are originated from chlorophyta species. For example, WRKY, a star transcription factor, used to be regarded as unique to land plants. However, in recent years, it has gradually become a mainstream view that WRKY is originated from chlorophyta (Chen et al., 2017).

### Gene Structure and Protein Domain

To obtain an insight into the structural diversity of CAMTA genes, the intron-exon organization was analyzed. The results showed that the number of introns in the CAMTA genes drastically varied, ranging from 0 to 34, but mainly concentrated between 10 and 13. In general, there were fewer introns in lower plants. These results were consistent with those of one previous study on a small number of plants (Rahman et al., 2016b). We also had some new findings when analyzing the phases of the introns. Phase pattern 0200-1102-0110 and 1102-0110-0200 were far more than other patterns in CAMTAs. Besides, the length of exons and the phase of introns exhibited a certain extent of correlation. For instance, the exons corresponding to 0200 or 0201 were generally significantly longer than those corresponding to 1102 or 0110. These results suggested that intron phase might be related to gene structure, but the possible reason for this phenomenon needs to be further studied.

Although CAMTAs contained a very conserved domain, we still observed the following gene structure variations. First, most CAMTAs contained TIG domain, but a small part did not. The distribution pattern of TIG domain in different phylogenetic clusters and species varied. Second, the length of CAMTA proteins varied greatly (745 ∼ 2309 aa), which might be mainly due to the different repetition numbers of ANK domains. Third, most CAMTA proteins had two IQ motifs (>70%), but some of them had one IQ motif (∼20%). In addition, very few CAMTA proteins contained three IQ motifs or no IQ motif. The variation of CAMTA domains indicated their functional differentiation. Further research and more evidence are needed to reveal the relationship between the protein structures and their functions.

### Calmodulin Binding Transcription Activator Expression and Its Function of Cold Resistance

The studies of CAMTA genes in the model plant *A. thaliana*, *O. sativa*, and *Z. mays* have generated abundant functional characterization data, which are valuable for functional prediction of their orthologous genes in other plants. CAMTAs have been reported to be related to maturity and development of plant organs in *P. tomentose* and *S. lycopersicum* (Yang T. et al., 2012; Wei et al., 2017). Based on these findings, we collected transcription data of a total of 43 tissues from *A. thaliana*, *O. sativa*, and *Z. mays* for further analysis. The results showed that the transcription level had significant tissue specificity and development stage specificity. Our analysis also indicated that CAMTA genes had the potential to regulate organ maturation and senescence.

In addition to modulating the maturation and development of organs and tissues, CAMTAs has also been found to play important role in cold stress response in *A. thaliana* (Doherty et al., 2009; Kim et al., 2013). *O. sativa* is very sensitive to low temperature stress, and it makes a series of physiological and metabolic responses under low temperature stress. However, most existing studies of cold stress-related genes in rice are limited to the preliminary mapping of quantitative trait loci (QTLs), and so far only 8 cold resistance-related genes have been cloned and identified (Mao et al., 2019). Especially, the function of CAMTAs in rice under cold stress remains largely unknown. We collected two sets of RNA-Seq data including PRJNA430015 and PRJNA607661 to investigate the roles of CAMTAs under cold stress. The intersection of DEGs detected by DESeq2 and edgeR and potential target genes detected by MEME was defined as target genes regulated by CAMTAs. A total of 255 CAMTA target genes were up-regulated, and 66 were down-regulated. Some down-regulated genes were involved in carbohydrate metabolism. Based on these results, we speculated that CAMTAs might improve the cold resistance of plants by regulating the genes related to carbohydrate metabolism to accumulate more carbohydrates. Some up-regulated target genes might be related to low temperature tolerance in *O. sativa*, of which transcription factor CBF1 is a case in point. The promoter region of CBF1 contains a CG-1 sequence at 1 kb upstream of the start codon. CAMTA has been confirmed to induce the expression of CBF1 under the cold stress in *A. thaliana* (Doherty et al., 2009), which is in line with our results that CAMTA also affected the expression level of CBF1 in rice, thus indicating the above cold-resistance mechanism may also be applicable to rice.

## Conclusion

In this study, we identified a total of 465 CAMTA genes from 112 plants. CAMTA gene was not detected in rhodophyta, but was identified from 6 out of 28 chlorophyta and all the evolutionarily young species. Therefore, we speculated that CAMTA might be originated from chlorophyta, which was also supported by a recent study that identified CAMTAs in *K. nitens* and *C. braunii*. We constructed a phylogenetic tree of CAMTA proteins based on the maximum likelihood, and inferred that CAMTAs might differentiate into three groups, and all these 3 groups expanded rapidly due to the great changes from aquatic to terrestrial environment. Gene structure and protein domain analyses indicated that the protein domains of CAMTA were basically conservative. However, the number of introns in the CAMTA genes dramatically varied, ranging from 0 to 34, and a certain extent of correlation was observed between the length of exons and the phase of introns. Gene expression profiles showed that the transcription level had significant species specificity, tissue specificity, and developmental stage specificity. The CAMTA genes presented the potential to regulate organ maturation and senescence. Under cold stress, CAMTAs in rice might regulate carbohydrate accumulation and target genes transcription, and these CAMTA genes work together with other transcription factors to resist low temperature stress. Our study provides a comprehensive insight into the origin and molecular evolution of CAMTA family and a valuable reference for further research on its biological functions.

## Data Availability Statement

The original contributions presented in the study are included in the article/Supplementary Material, further inquiries can be directed to the corresponding author/s.

## Author Contributions

JG and PX conceived, designed the research, and wrote the manuscript. PX conducted the identification, phylogenetic, and structure analysis. PX, J-WF, and X-TZ carried out the gene expression and functional analysis. All authors contributed to the article and approved the submitted version.

## Conflict of Interest

The authors declare that the research was conducted in the absence of any commercial or financial relationships that could be construed as a potential conflict of interest.

## Publisher’s Note

All claims expressed in this article are solely those of the authors and do not necessarily represent those of their affiliated organizations, or those of the publisher, the editors and the reviewers. Any product that may be evaluated in this article, or claim that may be made by its manufacturer, is not guaranteed or endorsed by the publisher.

## Footnotes

1
https://www.ncbi.nlm.nih.gov/

2
https://datadryad.org/stash/

3
https://www.fernbase.org/

4
https://www.arabidopsis.org/

5
https://wolfpsort.hgc.jp/

6
https://www.biobam.com/omicsbox/

7
https://itol.embl.de/

8
http://gsds.gao-lab.org/

9
https://www.ncbi.nlm.nih.gov/sra/

10
http://travadb.org/

11
http://jaspar.genereg.net/

## Supplementary Material

Supplementary Figure 1 Structure analysis of identified CAMTA genes.

Supplementary Figure 2 Phylogenetic tree and functional domains of identified CAMTAs. CG-1, CG-1 DNA-binding domain; TIG, transcription-associated immune globulin-like domain; ANK, ankyrin repeat domain; IQ, Ca^2+^-independent CaM-binding IQ motif; CaMB, Ca^2+^-dependent CaM binding domain.

Supplementary Figure 3 Expression level of CAMTA genes in 12 species.

Supplementary Table 1 A total of 112 plant genomes for subsequent analysis.

Supplementary Table 2 The transcription data for subsequent analysis.

Supplementary Table 3 A total of 465 identified CAMTA proteins.

Supplementary Table 4 Intron number and intron phase of identified CAMTA genes.

Supplementary Table 5 The selected representative species.

Supplementary Table 6 Length, molecular weight, and isoelectric point of identified CAMTA proteins.

Supplementary Table 7 Gene Ontology analysis of identified CAMTA genes.
