# Supplementary figures and images for: Evolution Analyses of CAMTA Transcription Factor in Plants and Its Enhancing Effect on Cold-tolerance
Source: Front Plant Sci. 2021 Nov 1;12:758187. doi: 10.3389/fpls.2021.758187 (PMC8591267; doi:10.3389/fpls.2021.758187)

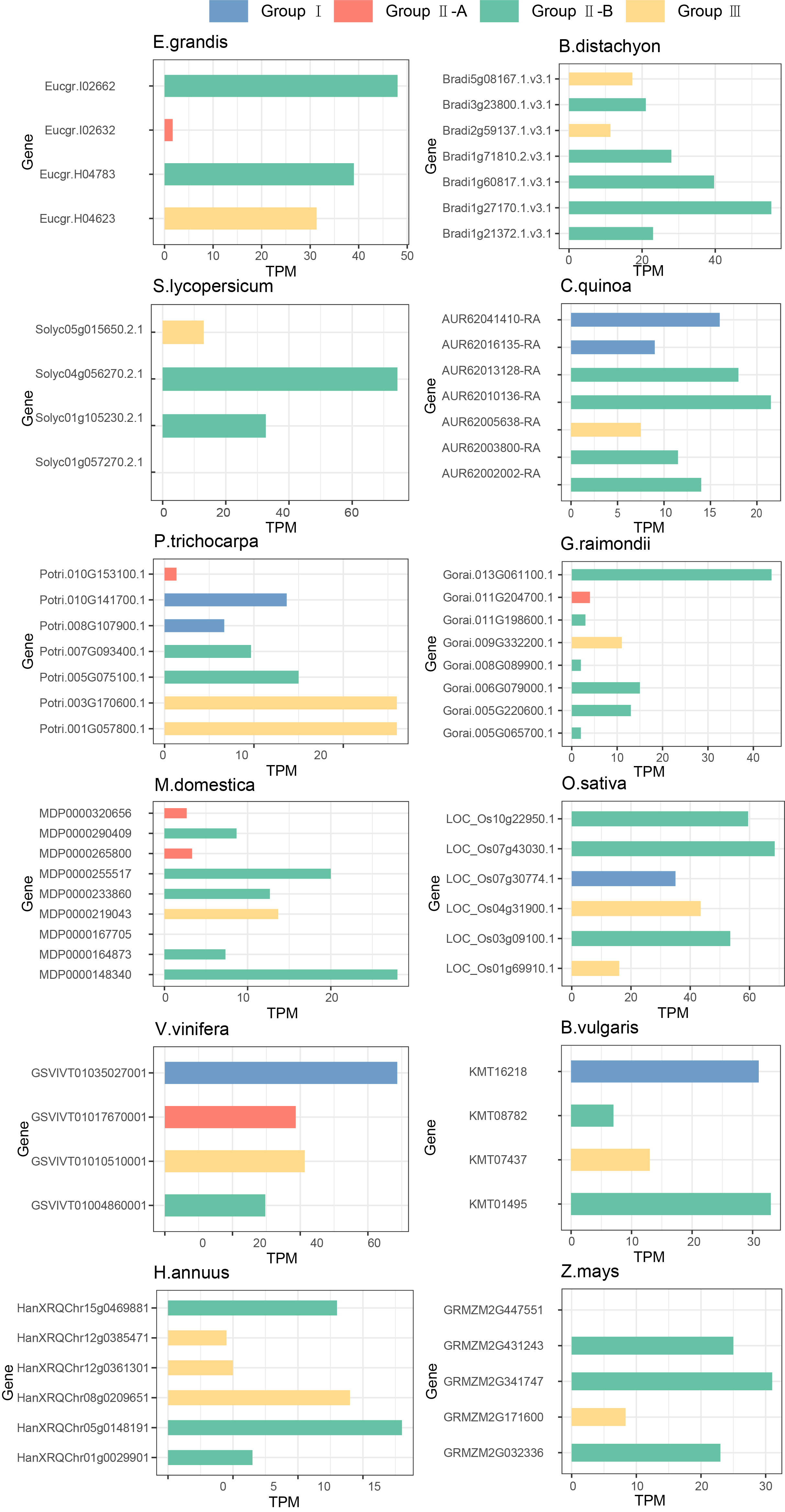

Supplement: Supplementary Figure 3 — Expression level of CAMTA genes in 12 species. [file Image_3.JPEG]
